# Awareness of HIV Status, Prevention Knowledge and Condom Use among People Living with HIV in Mozambique

**DOI:** 10.1371/journal.pone.0106760

**Published:** 2014-09-15

**Authors:** E. Kainne Dokubo, Ray W. Shiraishi, Peter W. Young, Joyce J. Neal, John Aberle-Grasse, Nely Honwana, Francisco Mbofana

**Affiliations:** 1 Division of Global HIV/AIDS, U.S. Centers for Disease Control and Prevention (CDC), Atlanta, Georgia, United States of America; 2 Division of Global HIV/AIDS, Centers for Disease Control and Prevention (CDC), Maputo, Mozambique; 3 Ministry of Health, Maputo, Mozambique; University of Buea, Cameroon

## Abstract

**Objective:**

To determine factors associated with HIV status unawareness and assess HIV prevention knowledge and condom use among people living with HIV/AIDS (PLHIV) in Mozambique.

**Design:**

Cross-sectional household-based nationally representative AIDS Indicator Survey.

**Methods:**

Analyses focused on HIV-infected adults and were weighted for the complex sampling design. We identified PLHIV who had never been tested for HIV or received their test results prior to this survey. Logistic regression was used to assess factors associated with HIV status unawareness.

**Results:**

Of persons with positive HIV test results (N = 1182), 61% (95% confidence interval [CI] 57–65%) were unaware of their serostatus. Men had twice the odds of being unaware of their serostatus compared with women [adjusted odds ratio (aOR) 2.05, CI 1.40–2.98]. PLHIV in the poorest wealth quintile were most likely to be unaware of their serostatus (aOR 3.15, CI 1.09–9.12) compared to those in the middle wealth quintile. Most PLHIV (83%, CI 79–87%) reported not using a condom during their last sexual intercourse, and PLHIV who reported not using a condom during their last sexual intercourse were more likely to be unaware of their serostatus (aOR 2.32, CI 1.57–3.43) than those who used a condom.

**Conclusions:**

Knowledge of HIV-positive status is associated with more frequent condom use in Mozambique. However, most HIV-infected persons are unaware of their serostatus, with men and persons in the poorest wealth quintile being more likely to be unaware. These findings support calls for expanded HIV testing, especially among groups less likely to be aware of their HIV status and key populations at higher risk for infection.

## Introduction

To achieve the vision of an AIDS-free generation, with no new HIV infections and no AIDS-related deaths [1], effective HIV prevention efforts are needed globally. In line with this, one of the goals of the Joint United Nations Programme on HIV/AIDS (UNAIDS) is to have 15 million people living with HIV (PLHIV), who are eligible for treatment, on antiretroviral therapy (ART) by 2015 [2]. Although a decrease in incidence has occurred in many low and middle-income countries [3], [4], HIV continues to pose a challenge to public health, especially in sub-Saharan Africa – the region most affected by the epidemic.

In Mozambique, with an estimated 1.4 million PLHIV and an adult HIV prevalence of 11.5%, HIV/AIDS is the second leading cause of death, accounting for 27% of all deaths in the country [3]. In collaboration with PEPFAR and other international and domestic partners, the government of Mozambique has been working to strengthen and sustain the national response to the HIV/AIDS epidemic [5]. The main objectives of the Mozambique National HIV Strategic Plan include reducing the level of new HIV infections and improving access to quality HIV treatment services for PLHIV.

Globally, most new HIV infections occur through sexual contact, and transmission occurs more commonly from persons unaware they have HIV, as PLHIV who are aware of their serostatus are more likely to increase their use of condoms during sexual intercourse and adopt behavioral changes to reduce the likelihood of HIV transmission [6]–[9]. Early initiation of ART reduces HIV transmission [10], [11] and is now an essential component of comprehensive prevention strategies. HIV testing is the entry point for individualized HIV care and treatment, and undiagnosed HIV infections undermine the effectiveness of HIV programs [12]–[15]. PLHIV should be diagnosed as early as possible after acquiring HIV infection, so that they can be linked to prevention and treatment services, and initiated on ART. The test-and-treat strategy for HIV prevention suggests that expanded HIV testing and early initiation of treatment could significantly decrease HIV transmission, and there are indications that the HIV epidemic could be lessened substantially by increasing the number of HIV-positive persons who are aware of their status [16]. In spite of the individual and public health benefits of HIV testing, a high proportion of PLHIV remain undiagnosed [17]–[20].

A comprehensive prevention strategy from HIV diagnosis to appropriate care and treatment for all PLHIV is needed, as well as a prioritization of services for PLHIV [4], [21]. To inform planning of effective HIV prevention programs, we assessed factors associated with HIV status awareness, HIV prevention knowledge and condom use among PLHIV in Mozambique using data from a large national HIV survey.

## Methods

We analyzed data from the 2009 National Survey on Prevalence, Behavioral Risks and Information about HIV and AIDS in Mozambique (Inquérito Nacional de Prevalência, Riscos Comportamentais e Informação sobre o HIV e SIDA em Moçambique), referred to as 2009 INSIDA.

### Ethics Statement

The survey was endorsed by the Mozambique Ministry of Health and received ethical approval from the Mozambique National Bioethics Committee for Health and the U.S. Centers for Disease Control and Prevention (CDC).

Written informed consent was obtained for all participants. For minors under age 18, consent was obtained from a parent or guardian, with assent requested from the minor. For emancipated minors (head of household or married) aged 15–17 years, written consent was obtained from the emancipated minor directly. Consent procedures were reviewed and approved by the IRBs.

### Survey Design

2009 INSIDA was an AIDS Indicator Survey, a cross-sectional nationally representative household-based survey that collected data on sociodemographic characteristics and HIV-related risk-behaviors, and included HIV testing for consenting participants [22]–[24]. The survey employed a conventional two-stage cluster sample design that was representative at the national, urban, rural and provincial level and that achieved relative errors of less than 0.3 for HIV prevalence among men and women aged 15–49 years in each province. The first stage involved selecting 270 census enumeration areas or clusters from both urban and rural domains. The second stage involved a systematic sampling of 22 households per urban cluster and 24 households per rural cluster, resulting in a total of 6,232 selected households [23], [24].

### Participants

A questionnaire was completed for selected households and captured information on all individuals in the household. Persons aged 12–64 years were eligible to participate in an individual interview and to provide a blood sample for HIV testing. Household questionnaires were completed in 99% of selected households. Among adults aged 15 – 64 years, 93% participated in the interviews and 92% were tested for HIV [22], [23].

### Laboratory methods

Capillary blood was collected from finger sticks from consenting individuals onto a filter paper. The dried blood spots (DBS) were then transported to the National Immunology Reference Laboratory in Maputo for testing and stripped of identifying information to ensure anonymity of the test. The laboratory protocol included an initial ELISA test (Vironostika HIV Uniform II plus O; bioMérieux, The Netherlands) and then retesting of all positive tests and five percent of the negative tests with a second ELISA (Murex HIV 1-2-O; Abbott Laboratories, UK). For those with discordant results on the two ELISA tests, a new ELISA (HIV-Genscreen HIV-1/2; BIO-RAD, France) was performed, with reactive tests classified as positive and non-reactive tests classified as negative [23], [25]. Because the testing was anonymous, survey respondents could not be provided with the result of the test. Instead, survey respondents were asked whether they wished to have home-based counseling and testing in order to learn their HIV status, and approximately two weeks after the survey team left each cluster, a community-based HIV counseling and testing (HCT) team visited the cluster and offered free counseling and testing to all survey participants who requested it and to others in the community who wished to be tested [23].

### Analyses

Our analyses focused on HIV-positive adults aged 15–49 years. We defined persons unaware of their HIV infection as those with a positive HIV test result in the survey, who reported that they had never previously been tested for HIV or were previously tested but never received their test result. All analyses were performed in SAS version 9.3 (SAS Institute Inc., Cary, NC, USA) using survey procedures (i.e., PROC SURVEYFREQ and PROC SURVEYLOGISTIC) to account for the complex design of the survey (i.e., clustering, stratification, and weighting). Domain analysis was used to assess subpopulations. Logistic regression analysis was conducted to examine associations between selected covariates and the outcome of interest, unawareness of positive HIV status.

We conducted a review of published research on knowledge of HIV status in sub-Saharan Africa and covariates included in our analysis were based on prior studies that explored factors associated with undiagnosed HIV infection. Selected sociodemographic factors including age, sex, place of residence, education level, wealth index and marital status were analyzed. In the survey, urban/rural designation was assigned based on whether or not a given enumeration area was located within a municipality; however it broadly reflects access to services such as sanitation, health and education. Wealth was defined using a Demographic and Health Survey standardized composite index of the living standard of a household, calculated using data on a household's ownership of selected assets (radio, television, bicycle, car, motorcycle), materials used for housing construction (roofing material, flooring and walls), access to drinking water and electricity, and sanitation facilities. Principal components analysis was used to develop weights for individual indicator variables, which were then combined into a continuous scale of household wealth, which was divided into quintiles, each with an equal number of households. Individuals were categorized according to the score of their household, ranging from the poorest to richest [26].

General HIV knowledge and sexual behaviors associated with HIV transmission were assessed in the survey. The survey assessed knowledge of HIV prevention methods – knowledge that HIV transmission can be prevented by abstinence, limiting sexual intercourse to a single uninfected partner, or use of condoms. We analyzed the data to determine if HIV prevention knowledge was associated with awareness of HIV-positive status. We also explored reported condom use at last sex – a validated measure of sexual risk behavior [27], to determine if condom use differed between PLHIV who were aware of their serostatus compared with those who were unaware of their HIV-positive status.

The following approach was used to construct the multivariable logistic regression model [28]. Variables with *p-*values less than 0.25 from single variable logistic regression analysis were included in the initial multivariable model. The multivariable model was refit until all variables in the model had *p-*values less than 0.05. Interactions between sex and all of the other variables in the final multivariable model were assessed; however, none of the interactions were statistically significant. Unadjusted and adjusted odds ratios and two-sided 95% confidence intervals (CI) are presented.

## Results

Of 9,030 (unweighted) adults aged 15–49 who were tested for HIV as part of 2009 INSIDA, there were 1,182 (unweighted) positive HIV test results, reflecting an HIV prevalence of 11.5% (95% confidence interval (CI) 10.3–12.6%) among Mozambican adults.

### Characteristics of PLHIV

In this national sample of persons in Mozambique, the median age was 29.6 years (interquartile range: 29.1–30.2). Among PLHIV, 65% (95% CI 63–68%) were female, 54% (95% CI 48–60%) resided in a rural area, 62% (95% CI 58–66%) had a primary education, 64% (95% CI 59–68%) were married or living with their partner, 70% (95% CI 66–74%) were currently employed, and 35% (95% CI 29–40%) fell in the richest wealth quintile.

### Awareness of HIV status

Among PLHIV identified in the survey, 673 (unweighted) (61%, 95% CI 57–65%) were unaware of their HIV infection, i.e. they reported that they had never previously been tested for HIV or were tested but never received their test result. Diagnosis information was missing for one person.

Reasons for not getting an HIV test were explored among PLHIV who had not previously been tested for HIV. Multiple responses were allowed, and the predominant reasons stated were that the individual did not feel ready to get a test (28% of respondents, 95% CI 23–33%), felt they were not infected (17%, 95% CI 13–20%), did not know where to go to get tested (17%, 95% CI 12–22%), did not perceive themselves to be at risk of getting AIDS (12%, 95% CI 9–15%), or were afraid to discover they were HIV positive (11%, 95% CI 8–14%). Other reasons not specified in the questionnaire were provided by 19% of respondents.

PLHIV who were aware of their serostatus were compared with those who were unaware of their HIV-positive status to determine correlates of HIV status awareness (Table 1). Men were more likely to be unaware of their serostatus relative to women, with an odds ratio (OR) of 1.78 (95% CI 1.31–2.43). After adjusting for age, wealth index and condom use, men were still more likely to be unaware of their serostatus with an adjusted odds ratio (aOR) of 2.05 (95% CI 1.40–2.98). Poverty had a significant association with HIV status unawareness in bivariate analysis (OR 3.08, 95% CI 1.15–8.24) and in multivariable analysis, PLHIV from households in the poorest wealth quintile were still more likely to be unaware of their serostatus (aOR 3.15, 95% CI 1.09–9.12) compared with those in the middle wealth quintile. PLHIV from households in the richer wealth quintile tended to be more likely to be aware of their serostatus, with an association that was significant in bivariate analysis (OR 0.59, 95% CI 0.35–0.97) but only marginally significant in multivariable analysis (aOR 0.61, 95% CI 0.35–1.07, *p* = 0.08). PLHIV from households in the richest wealth quintile were more likely to be aware of their HIV status (aOR for being unaware of status 0.37, 95% CI 0.22–0.65) than those in the middle wealth quintile.

**Table 1:** Characteristics of People Living With HIV by awareness of serostatus, Mozambique – 2009, [N = 1182].

	Unaware of serostatus *n* 1 (%)	Aware of serostatus *n* 1 (%)	OR (95% CI)	aOR^2^ (95% CI)
Age by 5-year groups				
15–19	57 (59.7)	29 (40.3)	1 [ref]	1 [ref]
20–24	105 (61.9)	86 (38.1)	1.10 (0.49–2.44)	0.92 (0.39–2.18)
25–29	144 (62.7)	119 (37.3)	1.14 (0.53–2.44)	0.90 (0.38–2.14)
30–34	113 (50.9)	119 (49.1)	0.70 (0.36–1.35)	**0.49 (0.24–0.99)**
35–39	103 (62.9)	68 (37.1)	1.14 (0.56–2.34)	0.68 (0.31–1.48)
40–44	79 (72.1)	39 (27.9)	1.75 (0.72–4.22)	1.17 (0.44–3.11)
45–49	72 (64.9)	43 (35.1)	1.25 (0.55–2.83)	0.97 (0.39–2.38)
Sex				
Male	261 (69.9)	118 (30.1)	**1.78 (1.31–2.43)**	**2.05 (1.40–2.98)**
Female	412 (56.6)	385 (43.4)	1 [ref]	1 [ref]
Place of residence				
Urban	336 (50.9)	350 (49.1)	1 [ref]	-
Rural	337 (69.9)	153 (30.1)	**2.24 (1.56–3.20)**	-
Highest education level				
No education	125 (70.5)	59 (29.5)	**4.33 (0.86–21.68)**	-
Primary	436 (64.3)	291 (35.7)	3.25 (0.66–16.10)	-
Secondary	109 (43.0)	145 (57.0)	1.36 (0.28–6.65)	-
Higher	3 (35.6)	8 (64.4)	1 [ref]	-
Wealth index				
Poorest	59 (89.5)	11 (10.5)	**3.08 (1.15–8.24)**	**3.15 (1.09–9.12)**
Poorer	89 (70.4)	31 (29.6)	0.85 (0.41–1.77)	0.82 (0.38–1.80)
Middle	103 (73.5)	44 (26.5)	1 [ref]	1 [ref]
Richer	211 (61.9)	132 (38.1)	**0.59 (0.35–0.97)**	0.61 (0.35–1.07)
Richest	211 (44.2)	285 (55.8)	**0.29 (0.17–0.48)**	**0.37 (0.22–0.65)**
Current marital status				
Never married	78 (60.1)	50 (39.9)	0.53 (0.15–1.80)	-
Married	53 (75.6)	28 (24.4)	1.08 (0.32–3.68)	-
Living together	371 (61.0)	273 (39.0)	0.54 (0.18–1.67)	-
Widowed	62 (57.5)	49 (42.5)	0.47 (0.14–1.54)	-
Divorced	22 (74.2)	6 (25.8)	1 [ref]	-
Not living together	87 (52.5)	97 (47.5)	0.39 (0.12–1.24)	-
Know that HIV can be prevented by abstinence				
No	129 (53.2)	130 (46.8)	0.76 (0.49–1.18)	-
Yes	404 (60.0)	310 (40.0)	1 [ref]	-
Know that HIV can be prevented by limiting to one sex partner				
No	70 (57.7)	58 (42.3)	0.930(0.57–1.50)	-
Yes	509 (59.5)	411 (40.5)	1 [ref]	-
Know that HIV can be prevented by use of condoms				
No	89 (67.4)	49 (32.6)	1.49 (0.91–2.43)	-
Yes	492 (58.2)	424 (41.8)	1 [ref]	-
Used a condom at last sex				
No	509 (66.6)	307 (33.4)	**3.01 (1.99–4.53)**	**2.32 (1.57–3.43)**
Yes	75 (39.9)	122 (60.1)	1 [ref]	1 [ref]

Note: The outcome for odds ratio (OR) and adjusted odds ratio (aOR) was modeled as unaware of HIV status.

1 All numbers presented are unweighted; percentages are weighted.

2 Adjusted for age, sex, wealth index and condom use.

### HIV prevention knowledge

Seventy-five percent (95% CI 72–79%) of PLHIV acknowledged that HIV can be prevented by being abstinent; 89% (95% CI 87–92%) stated that having a single sexual partner, and 86% (95% CI 83–88%) stated that using condoms during sexual intercourse can prevent transmission of HIV. We analyzed HIV prevention knowledge among PLHIV who were aware of their HIV-positive status and those who were not aware that they were HIV-positive to determine if there were differences between groups (Table 1). The knowledge that HIV transmission can be prevented by abstinence, having a single uninfected sex partner, and/or use of condoms was not significantly associated with awareness of HIV status.

### Condom use

In our analysis of HIV risk behaviors among PLHIV, specifically unprotected sex, 83% (95% CI 79–87%) of PLHIV reported not using a condom during their last sexual intercourse. Reasons for not using a condom were explored among PLHIV, and the most common reasons cited were that the respondent was married (51%, 95% CI 46–57%) and that they trusted their sexual partner (30%, 95% CI 25–34%). We analyzed condom use among PLHIV to determine if there were differences based on awareness of one's serostatus (Table 1). PLHIV who reported not using a condom during their last sexual intercourse were more likely to be unaware of their serostatus (OR 3.01, 95% CI 1.99–4.53) compared to PLHIV who reported using a condom. After adjusting for age, sex, and wealth index, not using a condom during last sexual intercourse among PLHIV was still significantly associated with being unaware of one's HIV-positive status (aOR 2.32, 95% CI 1.57–3.43).

## Discussion

In this nationally representative survey, 61% of adult PLHIV in Mozambique were unaware that they were HIV-infected. In a country where more than one in ten adults is living with HIV, the high proportion of persons unaware of their HIV status represents a substantial number of PLHIV who are not seeking care and treatment for their own health, and who are a potential source of new HIV infections.

Men represent 35% of PLHIV in Mozambique; however, they had almost twice the odds of being unaware of their HIV-positive status compared with women. Low rates of HIV testing among men have been observed in other high HIV prevalence settings [29]–[31]. These gender differences may be due to reduced health-seeking behaviors among men and also suggest that efforts to increase HIV testing and counseling have provided greater benefits to women. Integration of HIV and maternal and child health services target women of child-bearing age, and young women are likely to be captured during routine HIV testing in antenatal clinics.

Lower rates of HIV testing have been described among rural populations in comparison to those in urban settings [31]–[33] and suggest limited access to and/or uptake of HCT services in rural areas. In this analysis, place of residence was associated with HIV awareness in bivariate analysis, however, this association was not significant in multivariable analysis. Economic factors appear to play a role in HIV infection, with more than a third of PLHIV in Mozambique falling in the richest quintile. Recent population surveys also suggest higher HIV prevalence among wealthier individuals in countries in sub-Saharan Africa [34], [35]. Theoretically, persons with a higher socioeconomic status may have more sexual partners, putting them at increased risk for HIV. There also appears to be a socioeconomic gradient in awareness of HIV status, as persons in the poorest wealth index were most likely to be unaware that they were HIV-positive. The reverse was seen with PLHIV from households in the richest wealth quintile, who were most likely to be aware of their HIV-positive status. This association persisted even when adjusted for rural or urban residence and possibly reflects that current HIV testing and counseling services are not convenient or easily accessible to economically disadvantaged persons.

Among PLHIV who had not previously been tested for HIV, the main reasons for not testing centered on lack of readiness, low risk perception, and not knowing where to go to get a test. Perceived low risk of HIV infection is a major barrier to uptake of HIV testing and may undermine the benefits of increasing ART availability in sub-Saharan Africa. Individuals often assume that if they are currently abstinent, have a steady partner, are not part of a high-risk group, or do not have physical symptoms of illness, they are at low risk of infection [36]. A study on HIV risk perception and behavior among sex workers in Mozambique revealed misconceptions about HIV transmission and found that some sex workers did not see HIV as a major health risk [37]. There is a need for ongoing primary prevention interventions in Mozambique, aimed at raising awareness about how HIV is transmitted and how to reduce the risk of transmission. Increased HIV awareness would address individual factors that hinder testing by enabling persons to accurately assess their risk of acquiring HIV and motivating all persons to know their status. HIV testing services are of limited utility if persons are not aware of where to go to get tested. Ensuring widespread availability of HIV counseling and testing services and easy access to each location may increase uptake of HIV testing in Mozambique.

Prevention programs in Mozambique teach individuals how to avoid behaviors that increase their risks of acquiring HIV by promoting the ‘ABC’ approach of abstinence, being faithful or reducing the number of sexual partners, and consistent and correct condom use. Our assessment of knowledge of HIV prevention methods revealed that a majority of PLHIV had accurate knowledge that HIV transmission can be prevented by abstaining from sexual intercourse, having a single uninfected partner, or by using condoms during sex. Also, prevention knowledge was not associated with awareness of one's serostatus. However, this knowledge does not appear to have translated into action, as a vast majority of PLHIV reported not using condoms at last sexual intercourse, and this proportion is likely an underestimate due to social desirability bias. In addition, PLHIV who reported not using a condom at last sex were more likely to be unaware of their serostatus. This observation is in line with studies that have shown that HIV testing and counseling increases condom use [38], [39] and this would in turn decrease sexual transmission of HIV. Knowledge of one's HIV-positive status presumably leads to behavior change and results in PLHIV taking measures to reduce the spread of the virus to uninfected persons. HIV testing is thus an effective intervention in its own right and as part of a combination prevention strategy.

In 2009 INSIDA, survey participants were asked if they had ever been tested for HIV. In the analysis, a person who may have been tested five years ago and reported receiving their result was categorized as knowing their serostatus. Our case definition is based on the assumption that PLHIV who reported ever receiving test results in the past were aware of their HIV infection. It does not account for persons who may have received a negative test result but subsequently seroconverted, although this would result in an underestimate of the proportion of PLHIV who are unaware of their positive serostatus. HIV testing history and risk behavior are self-reported and subject to recall and social desirability bias, as individuals may be unwilling to admit to behaviors that put them at risk for HIV infection. In spite of these limitations, our findings indicate that awareness of one's HIV status is low among PLHIV in Mozambique, and persons who are unaware that they are HIV positive are less likely to adopt strategies to reduce HIV transmission to their sexual partners.

Not knowing one's HIV-positive status also limits an individual's ability to seek HIV-related care and early treatment initiation. Undiagnosed HIV infection is an obstacle to HIV prevention and treatment and can jeopardize both the individual's and the public's health. HIV counseling and testing services play an important role in HIV prevention by linking PLHIV to care and treatment services. In this era of increased HIV awareness, availability of HCT services and rapid HIV testing, every HIV-positive individual should know their status and be linked to care, for their own health and for the public's health. Efforts are needed to better understand deterring factors for HIV testing in Mozambique and appropriate steps should be taken to address them. Given limited resources, HIV testing and counseling programs need to use innovative ways to increase HIV testing among persons most at risk for HIV infection. Strategic expansion of HIV testing and counseling programs, targeted testing of populations at higher risk of infection and partners of PLHIV, as well as provider-initiated testing and counseling are strategies that should be employed in order to decrease the number of PLHIV who are unaware of their serostatus. Mozambique currently implements partner testing in antenatal clinics and couples testing in Community Health Counseling and Testing settings. Prevention strategies should also include the scale-up of HIV testing and counseling for couples to increase testing among individuals in regular or stable partnerships. Future AIDS indicator surveys can help identify trends in HIV testing uptake and prevalence of undiagnosed HIV infection among PLHIV. This analysis also reinforces the need for prevention interventions aimed specifically at HIV-positive individuals. To help break the chain of HIV transmission, prevention efforts should also focus on PLHIV to increase their awareness of how to prevent HIV, encourage their adoption of behaviors to reduce HIV transmission, and promote early initiation of HIV care and treatment.
